# Identification of aberrant tRNA-halves expression patterns in clear cell renal cell carcinoma

**DOI:** 10.1038/srep37158

**Published:** 2016-11-24

**Authors:** Malin Nientiedt, Mario Deng, Doris Schmidt, Sven Perner, Stefan C. Müller, Jörg Ellinger

**Affiliations:** 1 University Hospital Bonn, Department of Urology, Bonn, Germany; 2 Pathology of the University Medical Center Schleswig-Holstein, Campus Luebeck, Luebeck, Germany; 3 Research Center Borstel, Leibniz Center for Medicine and Biosciences, Borstel, Germany

## Abstract

Small non-coding RNAs (sncRNA; <200 nt) regulate various cellular processes and modify gene expression. Under nutritional, biological or physiochemical stress some mature sncRNAs (e.g. tRNAs) are cleaved into halves (30–50 nt) and smaller fragments (18–22 nt); the significance and functional role of these tRNA fragments is unknown, but their existence has been linked to carcinogenesis. We used small RNA sequencing to determine the expression of sncRNAs. Subsequently the findings were validated for miR-122-5p, miR-142-3p and 5'tRNA4-Val-AAC using qPCR. We identified differential expression of 132 miRNAs (upregulated: 61, downregulated: 71) and 32 tRNAs (upregulated: 13, downregulated: 19). Read length analysis showed that miRNAs mapped in the 20–24 nt fraction, whereas tRNA reads mapped in the 30–36 nt fraction instead the expected size of 73–95 nt thereby indicating cleavage of tRNAs. Overexpression of miR-122-5p and miR-142-3p as well as downregulation of 5'tRNA4-Val-AAC was validated in an independent cohort of 118 ccRCC and 74 normal renal tissues. Furthermore, staging and grading was inversely correlated with the 5'tRNA4-Val-AAC expression. Serum levels of miR-122-5p, miR-142-3p and 5'tRNA4-Val-AAC did not differ in ccRCC and control subjects. In conclusion, 5′ cleavage of tRNAs occurs in ccRCC, but the exact functional implication of tRNA-halve deregulation remains to be clarified.

Renal cell carcinoma (RCC) is the most frequent renal malignancy accounting for 80–85% of the primary renal tumors. The incidence of RCC is still increasing, especially the number of young patients and high-grade disease is rising^1^. The increasing number of small renal tumors may be explained by the widespread use of abdominal ultrasonography for check-up or clarification of non-specific symptoms. A substantial number of these small renal tumors turns out to be benign. Current imaging modalities do not allow precise identification of malignant tumors^2^, percutaneous biopsy has several limitations impeding the acceptance of the method^3^, and thus overtherapy is common as many renal masses are benign^4^. Thus, additional diagnostic parameters could help the clinician to improve patients treatment. Furthermore, small tumors are often growing slowly and active surveillance are alternative treatment options getting an increased acceptance in selected patients^5^. But, early identification of aggressive tumors is desirable as prognosis of advanced/metastatic RCC is poor: surgery (cytoreductive nephrectomy, metastasectomy) and targeted therapy improved patient’s survival, but eventually most patients decease as a consequence to the disease^6^.

Up until now, no biomarker is available for clinical practice, making an accurate and non-invasive identification of RCC impossible. Non-coding RNAs, especially small non-coding RNAs (sncRNA; <200 nt), have attracted the attention of biomarker researchers as sncRNAs act as a regulator of various cellular processes and may have oncogenic or tumor suppressive properties. miRNA, as a subclass of sncRNA, expression profiles have been established in RCCs^7^, and the detection of miRNAs in bodily fluids allows their use as non-invasive biomarker for patients with urological malignancies^8^. In contrast to miRNAs, few is known about the expression of the other sncRNA types, such as tRNA, sn(o)RNA and piRNA^9^. To improve the understanding of such interactions, we determined the expression profile of sncRNAs in clear cell renal cell carcinoma (ccRCC). We observed altered expression of truncated tRNA fragments in ccRCC. Among several deregulated tRNA, we identified 5′tRNA4-Val-AAC as downregulated in ccRCC, and furthermore its expression was correlated with advanced stage and grade.

## Results

### Small RNA expression profiling

**Figure 1:**
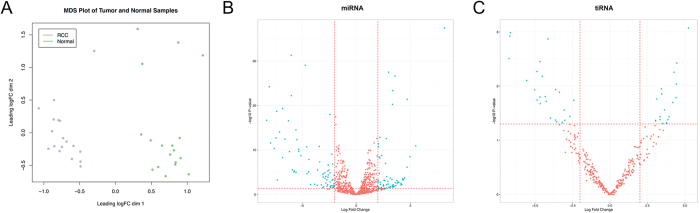
miRNA expression profiles discriminate normal and ccRCC tissue. (**A**) A multi dimensional scaling plot demonstrates accurate classification of 18 corresponding normal (green dots) and ccRCC (pink dots) tissue samples based on the miRNA expression profile. Distances between samples are corresponding to leading log2-fold changes between each pair of RNA samples. The leading log-fold-change is the average of the largest absolute log-fold-changes between the corresponding samples. The volcano plots are showing the expression of miRNA (**B**) and tRNA (**C**) in normal and ccRCC tissue. miRNAs/tRNAs with an at least 2-fold significant expression difference are indicated with blue dots.

sncRNA expression including miRNAs, tRNAs, piRNAs and sn(o)RNAs was profiled using small RNA sequencing. We investigated the sncRNA profile in a discovery cohort of 18 corresponding ccRCC and normal renal tissue samples. We observed differential expression (defined as log2-fold expression difference >2 and p-value < 0.05) of 132 miRNAs: 61 miRNAs were upregulated and 71 were downregulated in ccRCC. Many of these differentially expressed miRNAs have been reported before, but we also identified deregulated miRNAs not yet known to have a potential impact on ccRCC pathogenesis (e.g. miR-142-3p, miR-885-5p, miR-1910-5p, miR-186-3p, miR-4652-5p, miR-6737-3p, miR-508-5p, miR-513c-5p, miR-4485-3p, miR-513a-5p, miR-4461). A summary of the 10 most up- and downregulated miRNAs in ccRCC is provided in Table 1. As expected, miRNA expression profiles allowed precise discrimination of normal and ccRCC tissues: a multi-dimensional scaling plot identifies two clearly separable clusters of ccRCC and normal renal tissue samples, as shown in Fig. 1A. The volcano plot in Fig. 1B demonstrates the miRNA expression differences in normal and ccRCC tissue. A heatmap of miRNA expression in renal tissues is provided in Supplementary Figure S1.

**Figure 2:**
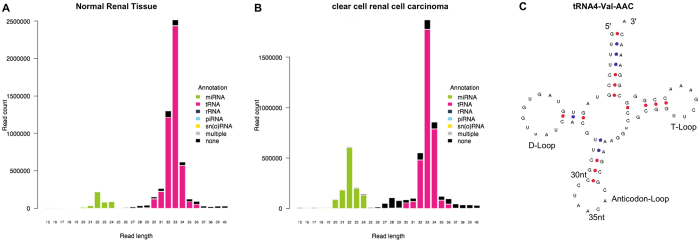
Read length distributions demonstrate the presence of tRNA cleavage. Read length distributions indicated the presence of two major peaks: a green peak indicates the enrichment of miRNA in the typical 20–24 nt fraction; a pink peak at 30–35 nt indicates the presence of 5′tRNA-halves. Exemplarily, the read length distribution of one corresponding pair of normal renal (**A**) and ccRCC (**B**) tissue is shown. It is important to note that the distribution of the read counts vary between the different corresponding samples. (**C**) the structure of tRNA4-Val-AAC is shown; it may be cleaved at the anticodon loop, resulting in 5′tRNA-halves of 30 to 35 nt, tRNA covariance model fold borrowed from Chan, P.P. & Lowe, T.M. (2009) GtRNAdb: A database of transfer RNA genes detected in genomic sequence. *Nucl. Acids Res.* 37(Database issue):D93-D97.

Beside the enrichment of miRNA, read length distribution analysis showed a second peak of RNA in the 30–36 nt part. Annotation analysis revealed that these reads derived from tRNAs (see Fig. 2). Among the 345 analyzed tRNA transcripts, we found 32 differentially expressed tRNAs: among these, 13 tRNA were upregulated and 19 were downregulated in ccRCC. The 10 most up- and downregulated tRNAs in ccRCC tissue are listed in Table 2. The differential tRNA expression is shown in a volcano plot in Fig. 1C. A heatmap of tRNA expression in renal tissues is provided in Supplementary Figure S2.

For a better understanding, gene wise expression and cluster analyses have been done using log2 normalized pseudo counts. Supplementary Figure S3 provides an overview about the homogeneity of gene expression of three selected targets. Comparable plots were observed for all significantly expressed miRNA or tRNA.

piRNAs were found in both tissue types, but only few of the sample reads did reach the small RNA sequencing normalization cutoff of 4 reads. Thus, most of them have not fulfilled the coverage of at least 1 counts per million (CPM) in each of two paired samples. Consequently, further analysis for these sncRNAs could not be performed. sn(o)RNA and rRNA-fragments were not found to be differentially expressed at all.

### Validation of small non-coding RNA expression

In order to confirm differential expression of selected sncRNAs we performed quantitative real-time PCR (qRT-PCR). The expression levels of 5′tRNA4-Val-AAC (small RNA sequencing: fold-change −6.648, p-value 0.0009, logCPM 19.473), miR-122-5p (small RNA sequencing: fold-change 8.148, p-value < 0.001, logCPM 5.101) and miR-142-3p (small RNA sequencing: fold-change 2.182, p-value < 0.001, logCPM 8.220) were studied in a validation cohort of ccRCC (n = 118) and normal (n = 74) renal tissue samples. As expected from the small RNA sequencing experiments, we noticed significant upregulation of miRNA-122-5p and miRNA-142-3p in ccRCC samples (p < 0.001). In contrast, miR-122-5p expression levels were decreased in metastatic ccRCC (p = 0.006). The expression levels of both miRNAs were not correlated with other clinicopathological parameters (pT-stage, lymph node metastasis, grade, age, sex; all p > 0.05).

**Figure 3:**
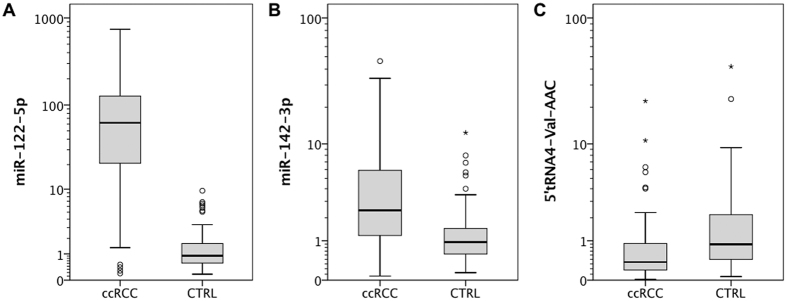
Validation of sncRNA expression using quantitative real-time PCR. The expression levels of 5′tRNA4-Val-AAC, miR-122-5p and miR-142-3p were different in ccRCC (n = 118) compared to normal (n = 74) renal tissue.

**Figure 4:**
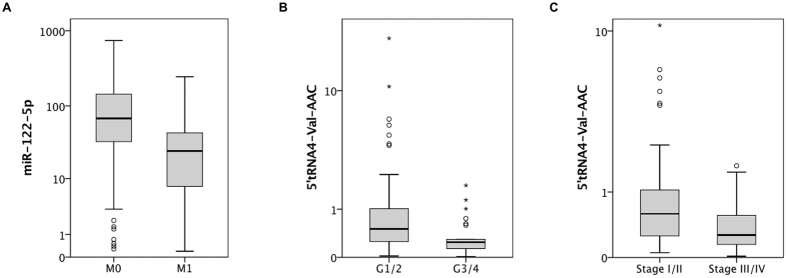
5′tRNA4-Val-AAC expression is associated with adverse pathology in ccRCC. Correlation of tissue small non-coding RNA levels with clinical-pathological parameters: (**A**) The expression of miR-122-5p was inversely correlated with M1-stage (p = 0.006), and the expression of 5′tRNA4-Val-AAC was inversely correlated with (**B**) less differentiated (p = 0.002) and (**C**) advanced (p = 0.001) ccRCC.

As shown in Supplementary Figure S4, we also performed qRT-PCR to determine the expression level of the full-length tRNA4-Val-AAC transcript in each 10 normal and ccRCC renal tissues. Interestingly, we did not notice any expression difference of the expression level between the normal and ccRCC tissue (p = 0.905). In contrast to this, we confirmed a significant decrease of 5′tRNA4-Val-AAC halves in ccRCC (p < 0.001). Also 5′tRNA4-Val-AAC levels were negatively correlated with tumor stage and grade: the decrease of 5′tRNA4-Val-AAC was more distinct in advanced (UICC stage III/IV vs. stage I/II: p = 0.001) and less differentiated (grade 1/2 vs. grade 3/4: p = 0.002) ccRCC. A boxplot figure indicating the expression differences is shown in Figs 3 and 4.

### Analysis of serum miR-142-3p, miR-122-5p and 5′tRNA4-Val-AAC levels

**Figure 5:**
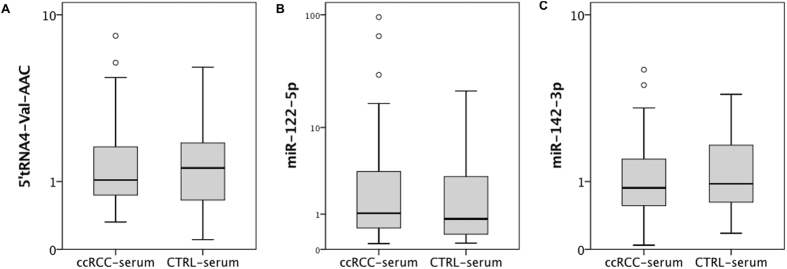
Analysis of sncRNA expression in serum. The expression levels of (**A**) 5′tRNA4-Val-AAC, (**B**) miR-122-5p and (**C**) miR-142-3p in serum samples are similar in ccRCC patients (n = 30) healthy control subjects (n = 15).

We also studied the expression of sncRNAs in serum samples obtained before nephrectomy to investigate the potential as non-invasive biomarker. However, we did not notice differential expression of miR-142-3p, miR-122-5p and 5′tRNA4-Val-AAC in serum samples (p > 0.05) in a cohort of 30 ccRCC patients and 15 healthy individuals. See Fig. 5.

### Functional analysis of miRNA-mRNA target interactions and relationships

We next used Cancerminer to evaluate functional miRNA-mRNA target interactions and relationships^10^. Interaction of miR-122 and mRNAs was predicted for CDCA7L (cell division cycle associated 7-like), CREG2 (cellular repressor of E1A-stimulated genes 2), IGLON5 (IgLON family member 5), FAM153C (family with sequence similarity 153, member C), LRRC10B (leucine rich repeat containing 10B), C3orf70 (chromosome 3 open reading frame 70), ARHGEF39 (Rho guanine nucleotide exchange factor (GEF) 39) and GRM5 (glutamate receptor, metabotropic 5. Notably, miRNA-122 is only expressed in glioblastoma multiforme and ovarian serous cystadenocarcinoma (REC-score between 2.13–3.16) besides of ccRCC within the TCGA dataset. Contrary to this miRNA-142 has a widespread expression in cancer tissue (REC-score between 2.09–16.93). It is known to be expressed in glioblastoma multiforme, ovarian serous cystadenocarcinoma, colon and rectal adenocarcinoma, lung squamous cell carcinoma, breast invasive carcinoma, uterine corpus endometrioid carcinoma, head and neck squamous cell carcinoma and lung adenocarcinoma. We identified 91 positive and significant mRNA target interactions. To mention the most important ones: CD2 molecule, ZNF831 (zinc finger protein 831), IKZF1 (IKAROS family zinc finger protein 1), GFI1 (growth factor independent 1 transcription repressor) and the XCL1 (chemokine ligand 1).

## Discussion

Despite of the numerous efforts to identify biomarkers for patients with RCC, there are currently none biomarkers available for daily practice. sncRNAs, especially miRNAs^11^, have been suggested as novel diagnostic/prognostic biomarkers. In order to increase the understanding and function on other sncRNA subtypes, we applied small RNA sequencing to sncRNAs and identified novel potential biomarkers for ccRCC.

The most interesting finding of our study is the identification of a large number (32 transcripts) of differentially expressed tRNA-halves, which has not been described before. We exemplarily validated downregulation of 5′tRNA4-Val-AAC in ccRCC using quantitative real-time PCR in an independent cohort of 118 ccRCC and 74 normal renal tissues. The potential relevance of this tRNA-halve is highlighted by the finding of the decreased 5′tRNA4-Val-AAC levels in patients with advanced stages and less differentiated ccRCC. Müller *et al*.^12^ evaluated a small RNA sequencing dataset (GSE24457) containing 10 ccRCC and normal samples, and thereby identified two downregulated tRNA derived fragments (tRNA-Leu-TTA and tRNA-Ser-TCA) in ccRCC^12^.

It was recognized that tRNA-halves circulate in a stable form in the bloodstream as particles of 100–300 kDA, but not in exosomes or other microvesicles^13^. tRNA-halves were subjected to regulation by age and calorie restriction^13^. Notably, various tRNA fragments in serum were circulating at different levels in breast cancer^14^ and head and neck squamous cell carcinoma^15^ patients compared to control subjects, suggesting a role as non-invasive cancer biomarker. We thus also investigated the levels of 5′tRNA4-Val-AAC in serum of ccRCC patients, but did not notice differential expression in a small cohort of ccRCC patients and healthy controls.

The read length analysis indicated that the differentially expressed tRNAs were truncated to 30 to 35 nt fragments, whereas mature tRNAs are 73 to 95nt sized. For a long time, tRNAs were solely known for their role in translation to decode nucleotide triplets for the protein synthesis. However, under nutritional, biological or physiochemical stress, but also under physiological conditions, mature tRNAs are cleaved into 5′halves (30–35 nt) and 3′halves (40–50 nt), as well into 5′tRFs and 3′tRFs (18–22 nt)^16^,^17^. In eukaryotes, the RNAse Angiogenin produces tRNA-halves through specific cleavage near or in the anticodon loop^18^. There is an increasing evidence that tRNA-halves have a functional role: 5′tRNA-halves inhibit translation for preservation of cellular energy under stress conditions^16^,^19^, protect cells from apoptosis by sequestering cytochrome c^20^ and induce angiogenesis^21^. They influence the formation of stress granules, which are targeting the translation initiation complex^19^. The involvement in key biological processes strongly suggests a functional role of tRNA-halves in RCC carcinogenesis. Their well-directed cleavage let us suggest that renal cancer cells are able to control the various tRNA-halve amounts quantitatively and qualitatively, which permits them a good and resistant stress adaptation. It was already demonstrated that sex hormone-dependent tRNA-halves enhance cell proliferation in breast and prostate cancer^22^.

miRNA expression has widely been studied in the past: tumor-specific miRNA expression profiles have been identified^23^, and miRNA profiles allow even specific identification of RCC subtypes^7^. The identification of circulating miRNAs in patients blood^24^,^25^,^26^ and their tremendous functional impact on carcinogenesis^9^ encouraged many researchers to study this RNA entity in ccRCC. In agreement with previous studies^7^,^12^,^27^,^28^,^29^,^30^, we were able to classify ccRCC and normal renal tissue based on the miRNome: we were able to detect 770 miRNA transcripts, and 17.1% (n = 132) were differentially expressed in ccRCC. In order to exemplarily validate the sequencing data, we determined the expression of miR-122-5p and miR-142-3p in an independent cohort of 118 ccRCC and 70 normal renal tissues. As expected, dysregulation of both miRNAs in ccRCC tissue was confirmed. An oncogenic function seems reasonable as miR-142-3p increased cancer cell proliferation through TGFβR1 repression in non-small cell lung cancer cell lines^31^ and activation of the WNT signaling pathway in breast cancer cells^32^. miR-122-5p was upregulated in primary renal tumors, but was observed downregulated during the metastatic process, a special finding also described by Wotschofsky *et al*.^33^ miR-122 acted in ccRCC cells as oncomir through activation of the PI3K/Akt signal pathway^34^ and was identified to target the VHL-HIF-hypoxia pathway^29^. Notably, circulating miR-122 facilitated metastasis by increasing nutrient availability in the premetastatic niche of breast cancer by inhibition of glucose uptake in normal cells^35^.

The class of piRNAs (26–32 nt) were identified 2006 in germline cells as a regulator of genomic stability; they take part in important cellular functions like transposon silencing, epigenetic regulation, proliferation and apoptosis. They have also been detected in variety of human somatic tissues^36^. Furthermore, it was shown that specific piRNA expression patterns in cancer cells exist^37^. However, our study does not support a role of piRNAs in ccRCC carcinogenesis.

Further on, several circulating miRNAs have been identified for some urological malignancies, like prostate cancer^38^, bladder cancer^39^ and RCC^26^. As miRNA tissue and serum expression profiles are often different, we investigated the expression of miR-122-5p, miR-142-3p and 5′tRNA4-Val-AAC-halve in an independent cohort of 45 serum samples. We observed that miRNAs as well as 5′tRNA-halves exist as circulating nucleic acids, but the expression levels did not differ in the ccRCC and the control cohort. This finding raises issues concerning the sncRNAs origin, their processing, their destination, their secretion mechanism, their transport and their target location. It seems possible that 5′tRNA-halves circulate in the bloodstream as part of a larger complex^13^ and miRNAs are suspected to be released passively and uncombined during tissue injury into serum or to be actively secreted in the circulation as microvesicles^40^. Therefore it is to determine whether does ccRCC change itself the sncRNA expression level and which extracellular functions do sncRNAs fulfill. Further, it remains unclear why the serum sncRNA expression profile differs from the sncRNA tissue profile. We expect that further studies can help to elucidate the sncRNA expression of ccRCC in bodily fluids and can shed light to the elusive molecular mechanism of sncRNA.

Some limitations of our study should also be acknowledged: we did not include technical replicates in our small RNA sequencing experiments, and thus data may have been misinterpreted. However, we ensured reliability by analyzing a large number of biological replicates (i.e. each 18 ccRCC and normal samples) in the sequencing experiment, and validation the finding of altered tRNA/miRNA expression in an independent, large-scaled validation cohort (118 ccRCC and 74 normal) using a different detection technique (PCR). It should also be noted that only 3 biomarkers were validated using PCR, although the expression of 132 miRNAs and 32 tRNAs was shown to be altered in ccRCC tissue. The expression differences in localized/advanced respectively low/high grade ccRCC seem to be small, although we observed a statistical significance; validation in an independent cohort is warranted. Functional analysis by computational methods enabled us to get an idea of the role of miR-122-5p and miR-142-3p in ccRCC development. Cancerminer use the huge molecular dataset from ‘The Cancer Genome Atlas’, so its results exhibit great empirical evidence. Nevertheless we should keep in mind, that bioinformatic data sources follow always a committed algorithm, which cannot cover all aspects of gene regulation and interactions.

## Methods

### Patients

Fresh frozen tissues from patients undergoing radical or partial nephrectomy were prospectively collected according standard operating procedures in the Biobank at the CIO Cologne-Bonn at the University Hospital Bonn. Samples were stored at −80 °C until usage. Renal tissue samples were collected between 1996 and 2014 from patients undergoing partial or radical nephrectomy for RCC; the samples were chosen randomly from the Biobank. All samples were reviewed using haematoxylin and eosin-stained sections by an experienced uropathologist (S.P.). RCC staging was performed according the 7^th^ edition of the TNM classification from 2009.

We also collected prospectively serum from ccRCC patients undergoing radical nephrectomy or partial nephrectomy as well as patients with non-malignant urological diseases between 2005 and 2011 at the Departments of Urology at the Universitätsklinikum Bonn (UKB). Blood was withdrawn preoperatively in Serum S-Monovette Gel tubes with clotting activator (Sarstedt, Nümbrecht, Germany). After clotting and centrifugation serum was separated and stored in cryotubes at −80 °C. The samples were processed within 3 hours.

All patients provided written informed consent and the study was approved by the Ethikkommission at the Universitätsklinikum Bonn (number: 280/12). All experiments were performed in accordance with relevant guidelines and regulations. The detailed clinical-pathological parameters of the study cohorts are reported in Table 3.

### RNA isolation

Total RNA was isolated using the mirVana miRNA Isolation Kit (Ambion, Foster City, CA, USA) and was two times DNAse-treated (DNA-free Kit, Ambion) according to the manufacturer’s recommendation. A detailed description was provided earlier^41^. RNA quality and quantity was measured (NanoDrop 2000 spectrophotometer, Thermo Scientific, Wilmington, DE, USA). RNA integrity of the samples was determined with a RNA 6000 Nano Kit on the Bioanalyzer 2100 (Agilent Technologies, Santa Clara, CA, USA); only samples with a RIN > 6 were used for small RNA sequencing experiments. Furthermore, all samples were investigated using agarose gel electrophoresis to exclude RNA degradation. Serum RNA isolation was performed as published earlier using the mirVana Paris-Kit (Ambion) from 400 μl serum^38^.

### Small RNA Sequencing

Small RNA sequencing was performed by Biogazelle (Zwijnaarde, Belgium) as a contract service. RNA isolates from 18 corresponding normal renal and ccRCC tissues (500 ng total RNA) were sent on dry ice to Biogazelle. Small RNA libraries were sequenced on an Illumina NextSeq500. Mapping was performed using the short-read aligner Bowtie (http://bowtie.cbcb.umd.edu). Bowtie is a free, open source software, which aligns ultrafast and memory-efficient Illumina reads to the human genome version 19. Mapped reads were subsequently annotated to different contaminents (tRNA, rRNA, sn(o)RNA, piRNA) and mature miRNA using Ensembl genome annotation database^42^, UCSC Genome browser^43^, miRBase v20^44^, and genomic tRNA database 2009^45^. Mismatches were not allowed. In case of multi-mapped reads, we assigned the reads to the sncRNA with the lowest offset. Supplementary Figure S5 demonstrates mean variance plots and scatterplots of miRNA and tRNA expression levels, used to ensure internal quality assurance.

Statistical analysis has been performed using the R programming language and the edgeR^46^ package following workflow proposed by Anders *et al*.^47^. Therefore, Counts Per Millions (CPM) have been calculated and sncRNA having less than 1 CPM in each sample pair been removed. This reduced the number of miRNAs from 2576 to 770 and of tRNAs from 624 to 345. For further analysis read counts have been normalized to the corresponding library sizes using edgeRs *calcNormFactors* function and sample variation has been taken into account by calculation the dispersion coefficients using the *estimateCommonDisp, estimateTagwiseDisp* functions. Detection of differentially expressed sncRNA has been performed using the exact test, as suggested by Robinson and Smyth and implemented edgeRs *exctTest* function^48^. Subsequently, a sncRNA has been called differentially expressed if its fold change was >2 or <(−2) and its p-value <0.05 (after Benjamini-Hochberg correction). To validate these finding a Generalized Linear Model (GLM) has been fitted to the data and detection of differentially expressed sncRNA has been done using the Likelihood Ratio Test (LRT), implemented as glmFit and glmLRT in the edgeR package, with both methods yielding identical results. Cluster and miRNA/tRNA wise expression analyses has been done using log2 normalized pseudo counts under consideration of the differentially expressed sncRNA from upstream operations. The raw data from small RNA sequencing experiments data are deposited at Gene Expression Omnibus (GEO) database (record: GSE73342)

### Real-Time PCR

To validate the expression profiling experiments, we determined the expression of three differentially regulated targets. PCR experiments were performed on an ABIPrism 7900 HT Fast Real-Time PCR System (Applied Biosystems, Foster City, CA, USA). qRT-PCR experiments were performed with an independent cohort of 118 ccRCC and 74 normal renal tissue samples. In addition, a serum cohort (30 ccRCC and 15 healthy individuals) was investigated to determine the value of sncRNA as non-invasive biomarker. Therefore, cDNA was synthesized with 500 ng RNA using the miScript II RT Kit (Qiagen, Hilden, Germany). Quantitative real-time PCR (qRT-PCR) was performed with 5 ng/μl cDNA (tissue) or 6 μl (serum) cDNA template using Qiagen miScript SYBR Green PCR technology (Hilden, Germany). A pre-designed miScript Primer Assay was used to quantify the reference gene SNORD43 (MS00007476) and the target gene miR-122-5p (MS00003416) and miR-142-3p (MS00031451); a custom made miScript Primer Assay (MSC0074992) was used to detect 5′tRNA4-Val-AAC. Data were analyzed using Qbase+ (Biogazelle) with SNORD43 as reference gene in the 2−∆∆CT algorithm^49^; target genes were scaled to the control group. Statistical analyses (Mann-Whitney-U test) were performed with SPSS Statistics v21 (IBM, Ehningen, Germany).

## Additional Information

**How to cite this article**: Nientiedt, M. *et al*. Identification of aberrant tRNA-halves expression patterns in clear cell renal cell carcinoma. *Sci. Rep.* **6**, 37158; doi: 10.1038/srep37158 (2016).

**Publisher's note:** Springer Nature remains neutral with regard to jurisdictional claims in published maps and institutional affiliations.

## Supplementary Material

Supplementary Information

## Figures and Tables

**Table 1:** Differential expression of miRNAs in ccRCC and normal renal tissue.

upregulated in ccRCC			
miRNA	log2-FC	p-value	logCPM
miR-122-5p	8.148	1.70E-36	5.101
miR-885-5p	5.500	3.33E-10	3.266
miR-1910-5p	4.995	5.06E-08	1.037
miR-592	4.703	2.26E-23	5.474
miR-3174	4.690	3.29E-07	−0.030
miR-186-3p	4.465	0.0003	−0.140
miR-4705	4.356	0.0010	−0.148
miR-4652-5p	4.299	0.0011	−0.174
miR-6737-3p	4.277	0.0004	−0.213
miR-885-3p	4.188	0.0212	−0.196
downregulated in ccRCC			
miR-508-5p	−8.283	1.87E-18	2.523
miR-184	−8.044	3.53E-24	3.993
miR-513c	−7.916	3.92E-12	2.220
miR-506-3p	−7.581	8.46E-11	1.914
miR-514a-5p	−7.407	1.45E-13	1.757
miR-508-3p	−7.355	7.83E-19	6.241
miR-4485-3p	−7.260	0.0054	3.346
miR-509-5p	−7.103	7.13E-14	2.587
miR-4461	−6.814	1.62E-19	2.613
miR-513a-5p	−6.804	2.04E-08	1.287

The logCPM value presents the mean log-average concentration/abundance of the 18 corresponding samples for each tag in the two groups being compared.

**Table 2:** Differential expression of tRNAs in ccRCC and normal renal tissue.

upregulated in ccRCC			
tRNA	log2-FC	p-value	logCPM
tRNA38_LeuCAG	5.251	0.0021	9.566
tRNA12_ProAGG	4.532	0.0096	8.494
tRNA8_ThrAGT	4.440	0.0068	7.399
tRNA86_ArgTCT	4.421	0.0161	7.387
tRNA164_MetCAT	4.274	0.0021	7.336
tRNA84_GlnTTG	4.206	0.0184	7.304
tRNA87_GluCTC	3.869	0.0418	16.177
tRNA2_LysTTT	3.644	0.0337	11.914
tRNA98_LeuAAG	3.517	0.0282	8.058
tRNA2_ProAGG	3.506	0.0291	7.452
downregulated in ccRCC			
tRNA9_ValCAC	−6.736	0.0037	17.899
tRNA18_ArgCCT	−6.684	0.0027	13.285
tRNA4_ValAAC	−6.648	0.0009	19.473
tRNA5_GluTTC	−5.560	0.0120	13.972
tRNA43_SerGCT	−5.125	0.0284	8.029
tRNA22_ProAGG	−4.875	0.0074	11.832
tRNA136_ValAAC	−4.864	0.0185	18.086
tRNA2_LeuTAA	−4.752	0.0120	8.086
tRNA6_ValCAC	−4.678	0.0185	15.873
tRNA128_GlyGCC	−4.655	0.0245	16.871

The used terms belong to the ‘genomic tRNA database 2009 legacy name and score’, following the human genome organization (HUGO) gene nomenclature committee guidelines (HGNC).

**Table 3:** Clinicopathological parameters of the study cohorts.

	screening	validation		serum cohort	
		ccRCC	CTRL	ccRCC	CTRL
	n = 18 (%)	n = 118 (%)	n = 74 (%)	n = 30 (%)	n = 15%
Sex					
male	13 (82.2)	81 (68.6)	52 (70.3)	18 (60.0)	7 (46.7)
female	5 (27.8)	73 (31.4)	22 (29.7)	12 (40.0)	8 (53.3)
Age					
mean	62.3	65.5	63.3	63.2	59.9
min-max	43–81	36–89	35–89	42–82	41–79
Pathological stage					
pT1	9 (50.0)	68 (57.6)	n.a.	15 (50.0)	n.a.
pT2	1 (5.6)	10 (8.5)	n.a.	0 (0)	n.a.
pT3	7 (38.9)	37 (31.4)	n.a.	15 (50.0)	n.a.
pT4	1 (5.6)	3 (2.5)	n.a.	0 (0)	n.a.
lymph node metastasis	0 (0)	4 (3.4)	n.a.	1 (3.3)	n.a.
distant metastasis	1 (5.6)	17 (14.4)	n.a.	1 (3.3)	n.a.
Fuhrman Grading					
grade 1	3 (16.7)	12 (10.2)	n.a.	1 (3.3)	n.a.
grade 2	13 (72.2)	81 (68.6)	n.a.	25 (83.3)	n.a.
grade 3	2 (11.1)	19 (16.1)	n.a.	4 (13.3)	n.a.
grade 4	0 (0)	6 (5.1)	n.a.	0 (0)	n.a.

n.a., not applicable.

## References

[b1] Torre L. A. . Global cancer statistics, 2012. CA: a cancer journal for clinicians 65, 87–108, doi: 10.3322/caac.21262 (2015). 25651787

[b2] Woo S. & Cho J. Y. Imaging findings of common benign renal tumors in the era of small renal masses: differential diagnosis from small renal cell carcinoma: current status and future perspectives. Korean journal of radiology 16, 99–113, doi: 10.3348/kjr.2015.16.1.99 (2015). 25598678 PMC4296282

[b3] Blute M. L. Jr., Drewry A. & Abel E. J. Percutaneous biopsy for risk stratification of renal masses. Therapeutic advances in urology 7, 265–274, doi: 10.1177/1756287215585273 (2015). 26425141 PMC4549697

[b4] Johnson D. C. . Preoperatively misclassified, surgically removed benign renal masses: a systematic review of surgical series and United States population level burden estimate. The Journal of urology 193, 30–35, doi: 10.1016/j.juro.2014.07.102 (2015). 25072182

[b5] Richard P. O. . Active surveillance for renal neoplasms with oncocytic features is safe. The Journal of urology., doi: 10.1016/j.juro.2015.09.067 (2015). 26388501

[b6] Ljungberg B. . EAU guidelines on renal cell carcinoma: 2014 update. European urology 67, 913–924, doi: 10.1016/j.eururo.2015.01.005 (2015). 25616710

[b7] Youssef Y. M. . Accurate molecular classification of kidney cancer subtypes using microRNA signature. European urology 59, 721–730, doi: 10.1016/j.eururo.2011.01.004 (2011). 21272993

[b8] Ellinger J. & Muller S. C. MicroRNAs: a novel non-invasive biomarker for patients with urological malignancies. Current pharmaceutical biotechnology 15, 486–491 (2014). 24846060 10.2174/1389201015666140519124909

[b9] Lee S. K. & Calin G. A. Non-coding RNAs and cancer: new paradigms in oncology. Discovery medicine 11, 245–254 (2011). 21447283

[b10] Jacobsen A. . Analysis of microRNA-target interactions across diverse cancer types. Nat Struct Mol Biol 20, 1325–1332, doi: 10.1038/nsmb.2678 (2013). 24096364 PMC3982325

[b11] Ellinger J., Muller S. C. & Dietrich D. Epigenetic biomarkers in the blood of patients with urological malignancies. Expert review of molecular diagnostics 15, 505–516, doi: 10.1586/14737159.2015.1019477 (2015). 25719388

[b12] Muller S. & Nowak K. Exploring the miRNA-mRNA regulatory network in clear cell renal cell carcinomas by next-generation sequencing expression profiles. BioMed research international 2014, 948408, doi: 10.1155/2014/948408 (2014). 24977165 PMC4054612

[b13] Dhahbi J. M. . 5′ tRNA halves are present as abundant complexes in serum, concentrated in blood cells, and modulated by aging and calorie restriction. BMC genomics 14, 298, doi: 10.1186/1471-2164-14-298 (2013). 23638709 PMC3654920

[b14] Dhahbi J. M., Spindler S. R., Atamna H., Boffelli D. & Martin D. I. Deep Sequencing of Serum Small RNAs Identifies Patterns of 5′ tRNA Half and YRNA Fragment Expression Associated with Breast Cancer. Biomarkers in cancer 6, 37–47, doi: 10.4137/bic.s20764 (2014). 25520563 PMC4260766

[b15] Victoria Martinez B. . Circulating small non-coding RNA signature in head and neck squamous cell carcinoma. Oncotarget 6, 19246–19263 (2015). 26057471 10.18632/oncotarget.4266 PMC4662488

[b16] Gebetsberger J. & Polacek N. Slicing tRNAs to boost functional ncRNA diversity. RNA biology 10, 1798–1806, doi: 10.4161/rna.27177 (2013). 24351723 PMC3917982

[b17] Anderson P. & Ivanov P. tRNA fragments in human health and disease. FEBS letters 588, 4297–4304, doi: 10.1016/j.febslet.2014.09.001 (2014). 25220675 PMC4339185

[b18] Yamasaki S., Ivanov P., Hu G. F. & Anderson P. Angiogenin cleaves tRNA and promotes stress-induced translational repression. The Journal of cell biology 185, 35–42, doi: 10.1083/jcb.200811106 (2009). 19332886 PMC2700517

[b19] Emara M. M. . Angiogenin-induced tRNA-derived stress-induced RNAs promote stress-induced stress granule assembly. The Journal of biological chemistry 285, 10959–10968, doi: 10.1074/jbc.M109.077560 (2010). 20129916 PMC2856301

[b20] Saikia M. . Angiogenin-cleaved tRNA halves interact with cytochrome c, protecting cells from apoptosis during osmotic stress. Molecular and cellular biology 34, 2450–2463, doi: 10.1128/mcb.00136-14 (2014). 24752898 PMC4054315

[b21] Fu H. . Stress induces tRNA cleavage by angiogenin in mammalian cells. FEBS letters 583, 437–442, doi: 10.1016/j.febslet.2008.12.043 (2009). 19114040

[b22] Honda S. . Sex hormone-dependent tRNA halves enhance cell proliferation in breast and prostate cancers. Proceedings of the National Academy of Sciences of the United States of America 112, E3816–E3825, doi: 10.1073/pnas.1510077112 (2015). 26124144 PMC4517238

[b23] Volinia S. . A microRNA expression signature of human solid tumors defines cancer gene targets. Proceedings of the National Academy of Sciences of the United States of America 103, 2257–2261, doi: 10.1073/pnas.0510565103 (2006). 16461460 PMC1413718

[b24] Wulfken L. M. . MicroRNAs in renal cell carcinoma: diagnostic implications of serum miR-1233 levels. PloS one 6, e25787, doi: 10.1371/journal.pone.0025787 (2011). 21984948 PMC3184173

[b25] Hauser S. . Analysis of serum microRNAs (miR-26a-2*, miR-191, miR-337-3p and miR-378) as potential biomarkers in renal cell carcinoma. Cancer epidemiology 36, 391–394, doi: 10.1016/j.canep.2012.04.001 (2012). 22542158

[b26] Wang C. . A panel of five serum miRNAs as a potential diagnostic tool for early-stage renal cell carcinoma. Scientific reports 5, 7610, doi: 10.1038/srep07610 (2015). 25556603 PMC5154588

[b27] Comprehensive molecular characterization of clear cell renal cell carcinoma. Nature 499, 43–49, doi: 10.1038/nature12222 (2013). 23792563 PMC3771322

[b28] Weng L. . MicroRNA profiling of clear cell renal cell carcinoma by whole-genome small RNA deep sequencing of paired frozen and formalin-fixed, paraffin-embedded tissue specimens. The Journal of pathology 222, 41–51, doi: 10.1002/path.2736 (2010). 20593407

[b29] White N. M. . miRNA profiling for clear cell renal cell carcinoma: biomarker discovery and identification of potential controls and consequences of miRNA dysregulation. The Journal of urology 186, 1077–1083, doi: 10.1016/j.juro.2011.04.110 (2011). 21784468

[b30] Osanto S. . Genome-wide microRNA expression analysis of clear cell renal cell carcinoma by next generation deep sequencing. PloS one 7, e38298, doi: 10.1371/journal.pone.0038298 (2012). 22745662 PMC3380046

[b31] Lei Z. . MiR-142-3p represses TGF-beta-induced growth inhibition through repression of TGFbetaR1 in non-small cell lung cancer. FASEB journal: official publication of the Federation of American Societies for Experimental Biology 28, 2696–2704, doi: 10.1096/fj.13-247288 (2014). 24558198

[b32] Isobe T. . miR-142 regulates the tumorigenicity of human breast cancer stem cells through the canonical WNT signaling pathway. eLife 3, doi: 10.7554/eLife.01977 (2014). PMC4235011 25406066

[b33] Wotschofsky Z. . Diagnostic and prognostic potential of differentially expressed miRNAs between metastatic and non-metastatic renal cell carcinoma at the time of nephrectomy. Clinica chimica acta; international journal of clinical chemistry 416, 5–10, doi: 10.1016/j.cca.2012.11.010 (2013). 23178446

[b34] Lian J. H., Wang W. H., Wang J. Q., Zhang Y. H. & Li Y. MicroRNA-122 promotes proliferation, invasion and migration of renal cell carcinoma cells through the PI3K/Akt signaling pathway. Asian Pacific journal of cancer prevention: APJCP 14, 5017–5021 (2013). 24175769 10.7314/apjcp.2013.14.9.5017

[b35] Fong M. Y. . Breast-cancer-secreted miR-122 reprograms glucose metabolism in premetastatic niche to promote metastasis. Nature cell biology 17, 183–194, doi: 10.1038/ncb3094 (2015). 25621950 PMC4380143

[b36] Ng K. W. . Piwi-interacting RNAs in cancer: emerging functions and clinical utility. Molecular cancer 15, 5, doi: 10.1186/s12943-016-0491-9 (2016). 26768585 PMC4714483

[b37] Martinez V. D. . Unique somatic and malignant expression patterns implicate PIWI-interacting RNAs in cancer-type specific biology. Scientific reports 5, 10423, doi: 10.1038/srep10423 (2015). 26013764 PMC4444957

[b38] Mahn R. . Circulating microRNAs (miRNA) in serum of patients with prostate cancer. Urology 77, 1265.e1269–1216, doi: 10.1016/j.urology.2011.01.020 (2011). 21539977

[b39] Scheffer A. R. . Circulating microRNAs in serum: novel biomarkers for patients with bladder cancer? World journal of urology 32, 353–358, doi: 10.1007/s00345-012-1010-2 (2014). 23266581

[b40] Brase J. C., Wuttig D., Kuner R. & Sultmann H. Serum microRNAs as non-invasive biomarkers for cancer. Molecular cancer 9, 306, doi: 10.1186/1476-4598-9-306 (2010). 21110877 PMC3002336

[b41] Blondeau J. J. . Identification of novel long non-coding RNAs in clear cell renal cell carcinoma. Clinical epigenetics 7, 10, doi: 10.1186/s13148-015-0047-7 (2015). 25685243 PMC4326488

[b42] Yates A. . Ensembl 2016. Nucleic acids research 44, D710–D716, doi: 10.1093/nar/gkv1157 (2016). 26687719 PMC4702834

[b43] Rosenbloom K. R. . The UCSC Genome Browser database: 2015 update. Nucleic acids research 43, D670–D681, doi: 10.1093/nar/gku1177 (2015). 25428374 PMC4383971

[b44] Kozomara A. & Griffiths-Jones S. miRBase: integrating microRNA annotation and deep-sequencing data. Nucleic acids research 39, D152–D157, doi: 10.1093/nar/gkq1027 (2011). 21037258 PMC3013655

[b45] Chan P. P. & Lowe T. M. GtRNAdb: a database of transfer RNA genes detected in genomic sequence. Nucleic acids research 37, D93–D97, doi: 10.1093/nar/gkn787 (2009). 18984615 PMC2686519

[b46] Robinson M. D., McCarthy D. J. & Smyth G. K. edgeR: a Bioconductor package for differential expression analysis of digital gene expression data. Bioinformatics (Oxford, England) 26, 139–140, doi: 10.1093/bioinformatics/btp616 (2010). PMC2796818 19910308

[b47] Anders S. . Count-based differential expression analysis of RNA sequencing data using R and Bioconductor. Nat Protoc 8, 1765–1786, doi: 10.1038/nprot.2013.099 (2013). 23975260

[b48] Robinson M. D. & Smyth G. K. Small-sample estimation of negative binomial dispersion, with applications to SAGE data. Biostatistics 9, 321–332, doi: 10.1093/biostatistics/kxm030 (2008). 17728317

[b49] Sanders I. . Evaluation of reference genes for the analysis of serum miRNA in patients with prostate cancer, bladder cancer and renal cell carcinoma. International journal of urology: official journal of the Japanese Urological Association 19, 1017–1025, doi: 10.1111/j.1442-2042.2012.03082.x (2012). 22788411

