# Supplementary material for: Identification of aberrant tRNA-halves expression patterns in clear cell renal cell carcinoma
Source: Sci Rep. 2016 Nov 24;6:37158. doi: 10.1038/srep37158 (PMC5121638; doi:10.1038/srep37158)

Supplementary Information

Title: Identification of aberrant tRNA-halves expression patterns in clear cell renal cell carcinoma

Authors: Malin Nientiedt; Mario Deng; Doris Schmidt; Sven Perner; Stefan C. Müller; Jörg Ellinger

Supplementary Figure 1

The heatmap indicates that normal (orange bar) and ccRCC (pink bar) tissues are accurately classified based on the miRNA expression levels.

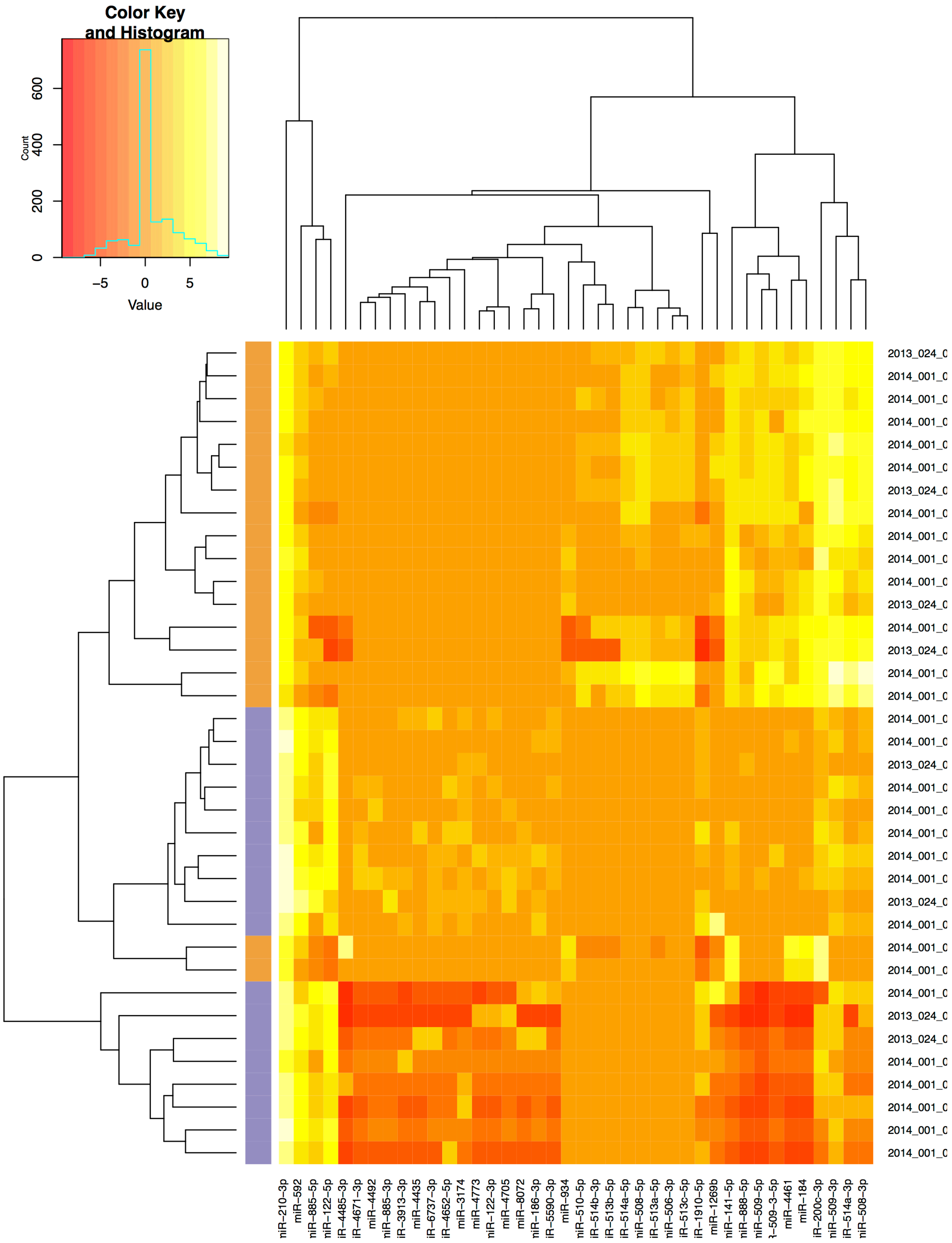

Supplementary Figure 2

The heatmap indicates that normal (orange bar) and ccRCC (pink bar) tissues are accurately classified based on the tRNA expression levels.

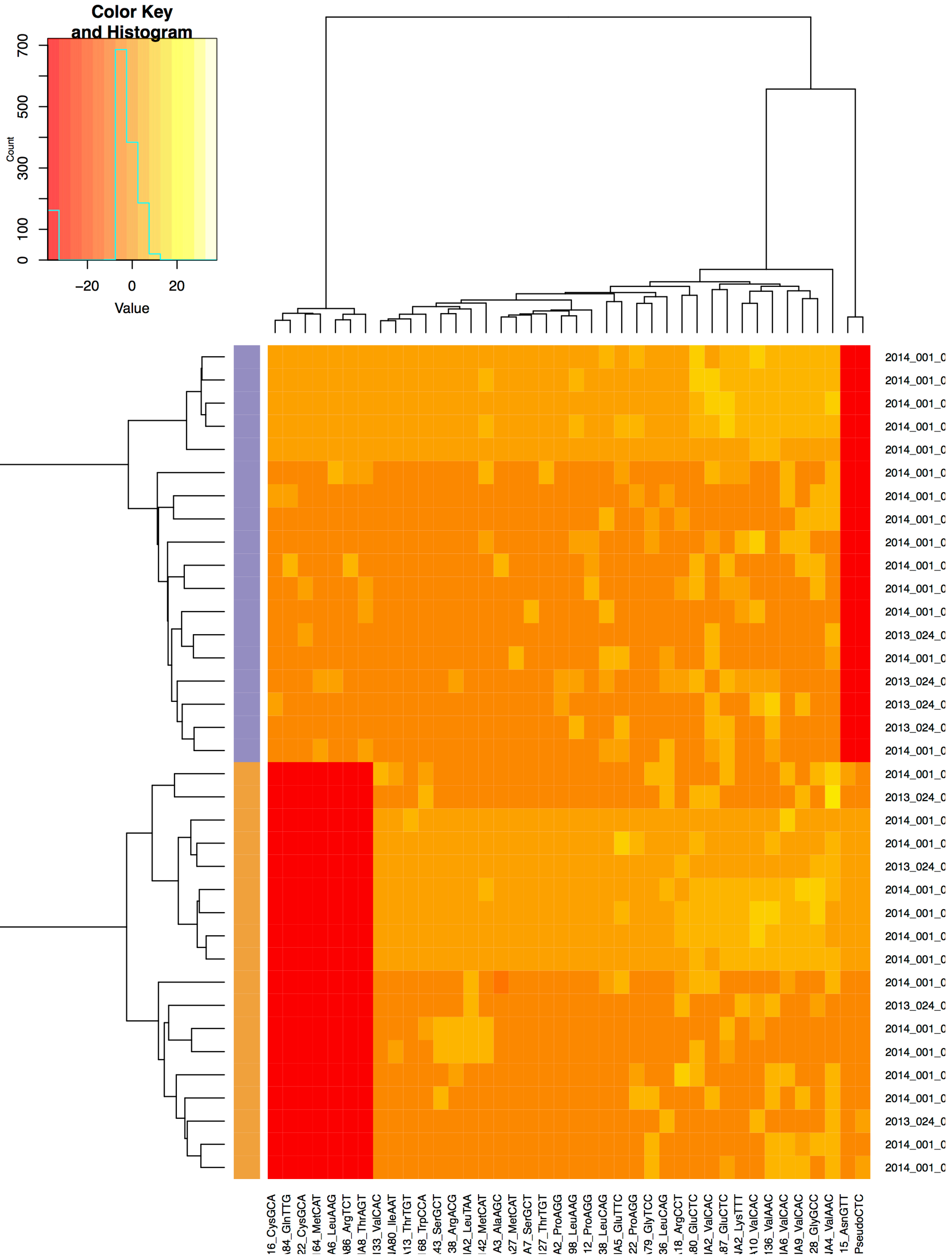

Supplementary Figure 3

Cluster and miRNA/tRNA wise expression analysis using log2 normalized pseudo counts under consideration of the differentially expressed sncRNAs from the small RNA sequencing experiments. The boxplot graphs demonstrate the distribution of 5’tRNA4-Val-AAC, miR-122-5p and miR-142-3p expression levels in normal (CTRL) and clear cell renal cell carcinoma (RCC) tissue.

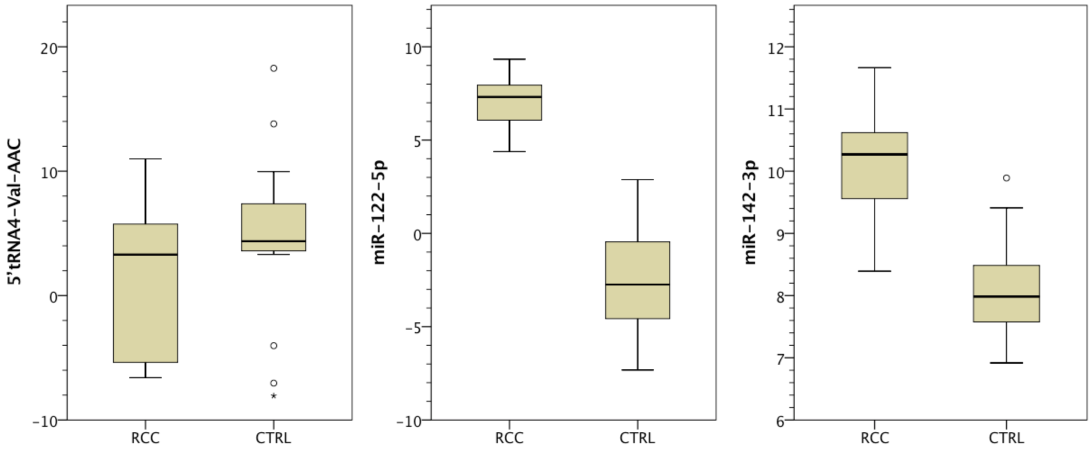

Supplementary Figure 4

Analysis of the full-length tRNA4-Val-AAC transcript: expression levels were similar (p=0.905) in the normal renal tissue (n=10) and clear cell renal cell carcinoma tissue (n=10) cohort.

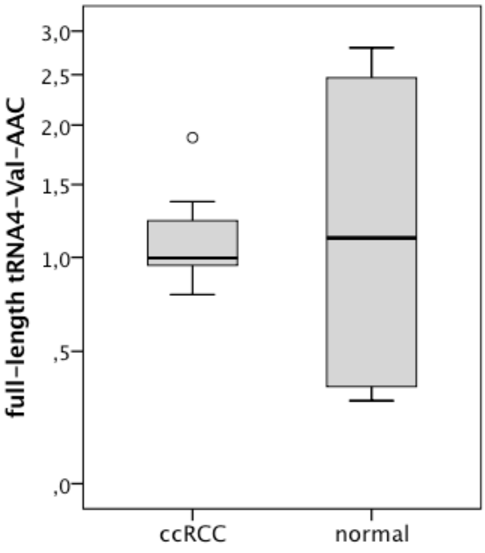

Supplementary Figure 5

Mean variance plots and scatterplots obtained in the small RNA sequencing experiments were used for quality assurance. The two mea variance plots at the top show the mean microRNA (left) and tRNA (right) expression levels for each gene in relation to its variance (including all samples). Red crosses demonstrate the raw data of reads, which are summarized in bins. Grey points are the variances of counts; blue points show the common tagwise dispersion estimate. The black line indicates the estimation of the statistical model.

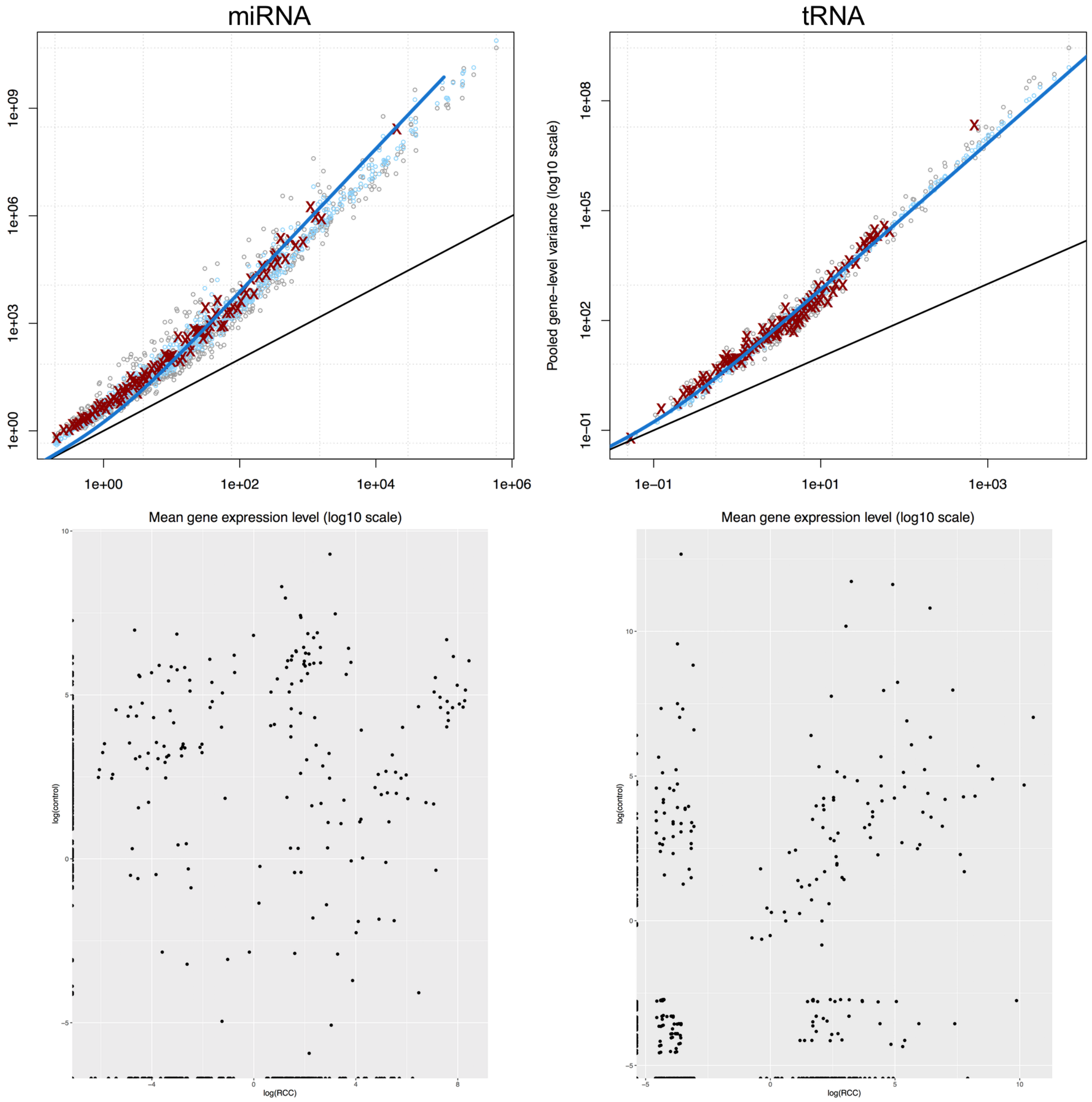

Supplement: Supplementary Information [file srep37158-s1.pdf]
